# Fluid teams in the metaverse: exploring the (un)familiar

**DOI:** 10.3389/fpsyg.2023.1323586

**Published:** 2024-01-10

**Authors:** Sirkka L. Jarvenpaa, Elizabeth Keating

**Affiliations:** ^1^Center for Business, Technology and Law, McCombs School of Business, The University of Texas at Austin, Austin, TX, United States; ^2^Department of Anthropology, The University of Texas at Austin, Austin, TX, United States

**Keywords:** metaverse, fluid teams, familiarity, TMS, virtual teams

## Abstract

The metaverse is a new and evolving environment for fluid teams and their coordination in organizations. Fluid teams may have no prior familiarity with each other or working together. Yet fluid teams are known to benefit from a degree of familiarity–knowledge about teams, members, and working together–in team coordination and performance. The metaverse is unfamiliar territory that promises fluidity in contexts–seamless traversal between physical and virtual worlds. This fluidity in contexts has implications for familiarity in interaction, identity, and potentially time. We explore the opportunities and challenges that the metaverse presents in terms of (un)familiarity. Improved understandings of (un)familiarity may pave the way for new forms of fluid team experiences and uses.

## 1 Introduction–the (un)familiar, the fluid, and the metaverse

Our environment, more than biology, shapes a vast majority of our conduct (Wilson, 1999). Our environment is a source of social learning and cognition. Socialization in a particular environment leads to familiarity with certain habits, tools, and skills and to expertly enacting them (Dewey, 1911).

This view of the importance of environment inspires questions about how the metaverse challenges and shapes the conduct of fluid teams. As a fully immersive, three-dimensional, multi-sensory environment, the metaverse and our socialization in it–in both social and technical processes–means becoming *familiar* with and accustomed to new habits, tools, and skills.

The metaverse has been defined as a 3-D, online environment where users represented by avatars “interact with each other in virtual spaces decoupled from the real physical world” (Ritterbusch and Teichmann, 2023).^1^ We take exception to this decoupled view, preferring instead an integrated, yet distinct, view of the metaverse. In this view, the metaverse comprises both virtual and physical environments that are integrated but distinct (Davis et al., 2009; Dwivedi et al., 2022; Marabelli and Newell, 2022; Mystakidis, 2022; Purdy, 2022; Hennig-Thurau et al., 2023; Richter and Richter, 2023). In any metaverse experience either the virtual environment or the physical environment can dominate.

In the metaverse, teams can collaborate, socialize, and communicate with flexibility, spontaneity, and psychological safety, drawing on resources from both physical and virtual environments (Davis et al., 2009; Purdy, 2022). In addition, team members might represent themselves as avatars or robots. Team members also might be artificial agents that are persistent and have continuity, independent of people. With the advances in digital technology, artificial agents are increasingly human-like and emotionally responsive (Etienne et al., 2023).

We expect the metaverse to be of great interest for the work of fluid teams. A fluid team comprises individuals who come together for a specific project or task and then disband once the project is completed or the task is accomplished (Reagans et al., 2005; Edmondson and Nembhard, 2009; Huckman et al., 2009; Bushe and Chu, 2011; Mortensen and Haas, 2018; Kerrissey et al., 2021). As such, membership is not stable, and members may have limited or varying experience in working with each other (Reagans et al., 2005; Edmondson and Nembhard, 2009; Huckman et al., 2009; Bushe and Chu, 2011; Mortensen and Haas, 2018; Kerrissey et al., 2021). Such teams may be very short-lived, such as flash teams (Valentine et al., 2017), or they may have a multi-year existence, as in engineering design teams (Keating and Jarvenpaa, 2016).

Fluid teams have resulted from the move to project-based work that cuts across various units in organizations and across broader ecosystems of organizations (Goetz et al., 2021). Fluid teams are now common across a wide variety of industries and physical and virtual settings, including health care (Kerrissey et al., 2021), education (Chávez-Miyauchi et al., 2021; Sandrone, 2022), gaming (Ching et al., 2021, 2022; Clement, 2023), emergency response teams (Majchrzak et al., 2007), software engineering (Huckman and Staats, 2011), and service operations (Valentine et al., 2019).

The fluidity of fluid teams arises from changing team membership, as well as from changes in work roles and work orientations and shifts in environment (Valentine and Edmondson, 2015). The lack of continuity–and thus of familiarity–differentiates fluid teams from other organizational teams. In the literature, familiarity often is seen as critical to communication and coordination success; aspects of familiarity include the duration of the shared work experience, the quality of the team relationships and cohesion between members, and the quality of team members’ communication (Muskat et al., 2022).

Fluid teams do rely on the familiar to structure the complexity and novelty they face (Valentine and Edmondson, 2015; Kerrissey et al., 2020), although the familiar is different from that of traditional organizational teams. For example, in fluid teams, familiarity might come from the information provided about team members and about organizational team role relationships (Goodman and Shah, 1992; Huckman and Staats, 2011). Familiarity thus would be low in a new fluid team and could strengthen as team members communicate and work with one another to frame and solve problems (Huckman et al., 2009) before the team disbands.

Some aspects of familiarity tend to be neglected in the literature on familiarity in teams. For example, shared environmental knowledge regarding cultural routines and behaviors often is taken for granted. How does lack of familiarity affect the organization of time, the understanding and use of shared space, aspects of self and identity, and human and virtual team member interactions? Such questions are particularly important in relation to familiarity, fluid teams, and working and coordinating in the metaverse. In this context, we find fluidity not just in the team membership, roles, tasks, and tools, but also as a consequence of moving between virtual spaces and physical places.

Familiarity also introduces challenges. Studies note that familiarity in physical face-to-face settings can reduce innovation and creativity (Zheng and Yang, 2015; Xie et al., 2020; Zaggl et al., 2022). Zhu and Yi (2023) argue that, in virtual environments, familiarity promotes an over reliance on habitual thinking and leads to inferior performance in a creative task. Familiarity also may reduce team members’ capacities for self-management and for shifting their direction as needed to handle unanticipated situations and to leverage spontaneity (Chávez-Miyauchi et al., 2021).

### 1.1 We ask the question: how does (un)familiarity play out in fluid teams in the metaverse?

Using existing research from anthropology, sociology, information systems, and organizational literatures, we consider familiarity in fluid teams in the metaverse from three perspectives: interactions, the self, and time. We focus on these three perspectives because our exploration of this work suggests that interpersonal *interactions* in the metaverse may at first seem familiar and recognizable based on prior socialization and culture. However, the virtual spaces of the metaverse allow users to enact *an extended self* and to engage in novel interactions, but without an adequate understanding of the risks that these extensions pose. The literature both on fluid teams and on the metaverse depicts *time* as a familiar cultural construct that synchronizes activities and can create habits and routines. However, time also can be manipulated and engineered in new ways in the metaverse. Thus, we suggest that time and temporal structures may represent something new and unfamiliar, especially as they relate to time–space relations for fluid teams in the metaverse.

## 2 What is the metaverse?

“Hype? Hope? Hell? Maybe all three.” Pew Research Center, 2022.

Etymology can be helpful in understanding the term *metaverse*. *Meta* is a prefix of Greek origin indicating after or beyond, as in *metaphysics* (Lee et al., 2022), or of a higher or second order, as in *metaverse*. It is beyond or a higher order of the physical world (i.e., the universe). Simply stated, the metaverse involves characteristics and functions that have not been possible before.

The term *metaverse* is attributed to Neal Stephenson, who developed the concept in his 1992 science fiction novel, *Snow Crash*. The main story in *Snow Crash* pits two rival teams in an epic power struggle over the fate of the real world and virtual world, both of which are threatened by a virus. The two teams include members from technical domains and various organizational entities, including Mafia bosses, teen-aged prodigy athletes, classical librarians, pizza delivery drivers, and software innovators. In relation to and as illustration of our focus in this paper, they come together to undertake a critical, time-limited task, just as fluid teams do. The characters also work together to solve mundane challenges, like getting a pizza delivered on time. The dynamic movement across worlds seems at first to be achieved simply by donning a set of goggles; but such movement also results from careful adjustments to new and unfamiliar ways to affect what happens next, as well as from being subject to a particular set of new or familiar rules (e.g., related to handshaking or hugs). In *Snow Crash*, individuals and teams constantly traverse physical and virtual spaces or environments; the worlds are primarily distinct, and yet also parallel and complementary.

This notion of the complementarity of physical and virtual worlds relates to philosopher Merleau-Ponty’s (1968) description of the human body: It is neither independent nor purely physical but is an entity that is always “in-between”; the body is constantly shaped and experienced through interactions with others and the environment and through language and gesture. In a similar way, the virtual and physical worlds can be separate for analytical purposes, taking the position of Suler (2016) that worlds can be distinct and separate–and yet they still interact.

In *Snow Crash*, in 1992, characters constantly invoke the *difference in the virtual world* from the real world. To illustrate, the Black Sun is a virtual nightclub that can be accessed and experienced by anyone who has the appropriate technology. It has its own unique atmosphere, characters, and music, unparalleled to anything that exists in the real world. People can visit the nightclub and interact with other users who also are present in the virtual space. It resides in a parallel virtual world that exists independently of the physical world. However, the worlds interact in that actions in the virtual world affect the real world, and vice versa. For example, Hiro, the main protagonist, is pursued by the virtual character Raven, who is sent to kill him. Hiro leaves the virtual world to enter the physical reality safe house, where he is protected from Raven’s attacks. Meanwhile, Hiro’s ally, Y. T., escapes from a dangerous situation in the real world, where she is being pursued by a group of thugs, by entering a virtual environment. She accesses her virtual goggles and enters the metaverse, where she is able to escape her pursuers and find a safe place to hide.

Part of what makes the fluid team of characters effective in *Snow Crash* is that the larger team comprises smaller teams (sometimes dyads), whose members first work together to address more mundane challenges–for example, timely pizza delivery, writing specific code, and protecting property. In the story, success depends on problem-solving skills, coordination, appreciation of novelty and diversity, creativity, access to accurate knowledge, physical stamina, egalitarian ethics, technical know-how, and self-maintained high standards of performance.

The novel inspired many Silicon Valley entrepreneurs and inventors to create a kind of reality from the ideas expressed first by Stephenson in narrative form. They borrowed Stephenson’s term, metaverse, to identify what they were trying to create in virtual spaces (Kelly, 2018). Bridging the fictional and the real worlds, Stephenson (1992), p. 240 notes that the virtual architecture “is made out of code. And code is just a form of speech–the form [language] that computers understand”.

Drawing on Stephenson’s early work, we focus on the currently evolving state of the metaverse as a collaborative environment for team members and fluid teams. We also note that the definitions and understandings of the virtual environment of the metaverse are as much *in the future* as they are in the present. With the advances in digital technology, the virtual environment becomes increasingly immersive and engaging (Seidel et al., 2022; Hennig-Thurau et al., 2023; Richter and Richter, 2023). In addition, virtuality allows for the metaverse’s massive, synchronous, and scaled character, as well as data persistence. Data persistence makes possible identity, history, entitlements, objects, and communications. Note that advancing technologies already often associated with the metaverse include advanced communications, virtual and augmented reality, Internet of Things, digital twins, haptic technology, and artificial intelligence (Park and Kim, 2022).

Organizational applications for the metaverse, both as a concept and a novel environment, are being widely explored. Some researchers have explored how the metaverse affects industrial and administrative processes related to education and training (Dwivedi et al., 2022; Purdy, 2022). Other research considers collaboration, innovation development, design activities, production, and quality control (e.g., Lee et al., 2022; Zhu et al., 2023). The metaverse has been deployed in surgical operations (Kelly, 2018), Alzheimer’s disease care (Purdy, 2022), and religious services (Whang and Miranda, 2023).

We recognize digital duality in the metaverse. Digital duality, which assumes the separation of real and virtual worlds, is more an analytical distinction than one observed in practice. Its relevance primarily arises when discussing futuristic adaptations. In addition, this distinction commonly is used when people refer to their activity spaces: For example, they typically distinguish time online from time offline; in addition, engaging in offline, physical world interactions does not require the new skills and habits and adaptation to innovations that online interactions require. Despite this common decoupling of physical and virtual worlds, many scholars recognize that the offline and online worlds now are impossible to separate: “[D]igital and material realities dialectically co-construct each other” (Jurgenson, 2009).

Below we clarify challenges to fluid teams, especially in relation to familiarity. Familiarity is considered by many metaverse designers a key to adaptability in the metaverse, yet familiarity has both benefits and constraints. We then discuss the metaverse, fluidity and familiarity in terms of interaction, the self, and time, creating new challenges for team coordination.

## 3 Fluid teams, team coordination, and familiarity

Researchers have examined fluid teams in both physical, face-to-face environments and virtual environments, although mostly separately. In both cases, the fleeting nature of the team membership arises because of a set of emerging diverse knowledge needs (Rulke and Galaskiewicz, 2000), the limited supply of skilled members (Espinosa et al., 2007), or emergent events, such as the loss of a member because of an injury (Pasarakonda et al., 2023). Fluid teams are commonplace across a wide variety of contexts, including healthcare, software engineering, transportation crews, and emergency and crisis teams.

Team coordination is paramount for fluid teams because of interdependencies in tasks (Olabisi and Lewis, 2018; Bachrach et al., 2019). However, the unbounded nature of memberships, relationships, locations, and time presents challenges. Complexities in coordination also arise from emergent differences that deviate from members’ prior experiences (i.e., from what is familiar to them) and from requirements that contract or expand team boundaries (Mayo, 2022). Fluid teams allow for working with different partners both in peer and hierarchical relationships, which can increase learning not only through meaningful discussions about unresolved issues but also through exposure to diverse partners (Akşin et al., 2021; Kim et al., 2023).

When fluid teams are most successful, studies have attributed this success in part to skillful management of transactive memory systems (TMSs). TMSs are shared systems for encoding, storing, and retrieving information (Wegner, 1995; Lewis, 2004; Majchrzak et al., 2007). Colloquially described as “knowing who knows what,” TMSs allow project members to locate expertise and to improve coordination–and hence, trust–when engaging in complex and interdependent tasks in a tight timeframe (Argote and Ren, 2012). As mechanisms for coordination, TMSs focus on precise and up-to-date knowledge of differentiated expertise structures to manage task interdependence (Olabisi and Lewis, 2018; Bachrach et al., 2019). Thus, they allow fluid teams to avoid coordination that depends on members’ shared understandings of their beliefs, preferences, goals, and strategies (O’Toole et al., 2023).

Research on fluid teams has found that, in rapidly changing environments, well-developed TMSs allow team members to trust in the knowledge received, particularly when they have no time to verify information and ideas (O’Toole et al., 2023). As task conflicts emerge, TMSs enable team members to address them in ways that enhance fluid team innovation–as long as relationship conflict has not also emerged (O’Toole et al., 2023). Research shows that team members who have shared and worked together on complex cases develop TMSs more effectively, thus emphasizing the benefits of familiarity; however, these team members also are able to coordinate better in future cases (Avgerinos and Gokpinar, 2017). TMSs facilitate team coordination because they can free up scarce cognitive resources; members do not have to exert mental effort on the tasks of others, and freed resources promote greater team adaption to novel problems. Therefore, TMSs at the team level are beneficial not just as task and social support but also for individual creativity (He et al., 2022).

Yet, the temporary nature of fluid teams contributes to the challenges of developing and accurately updating the TMSs that can support them (Jarvenpaa and Keating, 2011). These challenges are exacerbated when a fluid team is dispersed geographically and temporally (Bachrach et al., 2019). In addition, the challenges exist in both physical face-to-face and virtual environments (see, e.g., Kanawattanachai and Yoo, 2007). In virtual environments, members face challenges in developing accurate perceptions of task-related expertise, shared views of where the expertise is located, and the ability to validate the expertise (Majchrzak et al., 2007). A shared context in which fluid teams can develop TMSs may be completely lacking. In *Snow Crash*, dyad structures are important in building TMSs but also that TMSs can include familiar non-human agents and mechanical creatures. In fact, much of the updating of TMSs in the story is supported by or delegated to artificial agents that compile and update information and act as an important resource for team members. Lack of familiarity with agents or environment renders knowledge transfer difficult and institutional knowledge hard to preserve, which can severely undermine team effectiveness.

Familiarity has been seen as an advantage in both fluid teams and traditional teams. Muskat et al. (2022) summarized the advantages of familiarity in work teams: Enhanced communication, learning, adaptation to change, and consensual decision making arise at the team level, while enhanced efficiency and effort reduction operate at the organizational level, serving to reduce complexity and minimize effort by allowing teams to draw on routines and on what they already hold in memory. Anthropologists and philosophers have long drawn attention to the importance of familiar (and often unconscious) habits that are widely shared and physically expressed in the body and the spaces and objects around it (Dewey, 1911; Bourdieu, 1977). These habits structure approaches to future tasks, and the future is materialized in present innovations and ideas (Vygotsky, 1978). As familiarity maps to existing mental routines and shortcuts, actors have less need to consider multiple possible responses to determine the most appropriate one (Dixon et al., 2014). As they spend less time on adaptation and hence on effortful processes, the result is greater immersion, ease of use, and enjoyability. Given these benefits, we might be surprised at how little is known about “*how long it takes to get familiar*” and “*how time is filled*” (Muskat et al., 2022, p. 1).

Constraints of the familiar also are significant. They include the risk of groupthink and the suppression of new ideas or constructive criticism to preserve harmony; resistance to change or to new team members; stagnation and complacency; and limits to innovation and adaptation to new challenges. An absence of familiarity may provide benefits by preventing social routines from becoming overly rigid, and members’ lack of familiarity can be a powerful basis for dialogic coordination (Zaggl et al., 2022).

## 4 The metaverse and familiarity

The familiar, or representations of it, have been intentionally used in designing user interfaces that conceal the complex computer code that makes virtual environments possible. In *Snow Crash*, Stephenson cites the importance of Apple Computer’s encouragement to programmers to “take advantage of people’s knowledge of the world around them by using metaphors to convey concepts and features of your application”; this mimicry would allow users to leverage an existing “set of expectations to apply to computer environments” (Kelly, 2018, p. 74). In contrast to IBM’s dos commands, Apple created interfaces based on innovations at Xerox Parc, which mapped prior experiences and items (e.g., recycling bins) onto computer screens (Dix et al., 1998).

Users in virtual environments encounter familiar structures that remind them of the “real world”–conventionalized, metaphorical representations that recruit the familiar in the service of the yet unknown. Metaphors work by proposing a relation; they are widely used in science to make complex concepts accessible to non-specialists–for example, the familiar and now conventionalized solar system model of the atom or the billiard ball model of gasses. These correspondences serve as tools for understanding our world and ourselves in it, structure our understandings of life, and have persuasive power (Lakoff and Turner, 2009, pp. xi–xii).

Designers of virtual metaverse objects similarly use forms that would be familiar to users as they move about in the physical world, designing representations of roads, buildings, grass, sky, and vehicles. In Mark Zuckerberg’s video, “Everything Facebook revealed about the Metaverse in 11 min”,^2^ he stands in a living room with a view of a tropical bay and says you can have a room of whatever you find most beautiful; in other words, you bring familiar preferences for space and views to Zuckerberg’s metaverse. One of the themes of *Snow Crash* is the team members’ acceptance of the illusion in the metaverse, which serves to partly convey that “everything is as expected.”

In developing guidelines to assist in effective socialization in an organizational metaverse, Gräf et al. (2023) emphasize the necessity of familiarity in virtual objects to help with perception. Said (2023) notes that “[t]he design needs to be simple and comprehensive to avoid learners’ cognitive overload. It also is to be consistent with the real world, in order to minimize the uneasiness that could be caused during switching between two realities.” Research finds that virtual meeting technology continues to face adoption challenges in organizations (Abramczuk et al., 2023). This hesitance is attributed to the high mental load even for completing mundane tasks (Xi et al., 2023), although the mental load depends on which automated tools are available (Jeffri and Rambli, 2021). Distractions when others are present in the metaverse also make complex tasks harder to complete (Marx et al., 2022).

Scholars have studied AI agents and familiarity (Ahn et al., 2021, 2022; Li and Sung, 2021; Baek et al., 2022). Studies assume that artificial agents can behave in ways that are reliably similar to how humans behave (Fiore and Wiltshire, 2016; Jarrahi, 2018; Csaszar and Steinberger, 2022).^3^ In one design, a record of each artificial agent’s experiences was connected to a large AI language model, giving the artificial agent the ability to synthesize its memories over time into higher level reflections and then to retrieve them, a result similar to human planning behavior. Metaverse platforms like Roblox and Zepeto provide human-like avatars, including the ability to express vivid emotions that replicate the person’s real-world appearance and behavior. This type of emulation of the physical and functional aspects of the real world has been found to engender trust and favorable attitudes (Suh et al., 2011). The goal of programmers’ agent design was to create believability in agent behavior, an “illusion of life,” and a “facade of realism” (Park et al., 2023).

However, trade fair interactions show that participants’ autonomy and freedom of choice, more than realism, were decisive factors for the success of an interactional event in virtual environments (Böhm and Müller, 2022). Avatar attendants’ attention spans in virtual environments were fragile unless users had multiple options to explore; they became disengaged when faced with the simulated familiar. In addition, educators note the necessity of unfamiliarity in learning; next-generation virtual classrooms are imagined as fluid contexts that might entail teleporting from a history class in Ancient Greece to an astronomy class held on Mars within a single platform.^4^

The question, then, is whether a focus on familiarity–as a way to combat complexity and introduce simplicity–causes metaverse designers and users to lose opportunities to explore and collaborate. For example, language provides an architecture, and using this structured architecture, people are extraordinarily creative in generating new ideas in what might seem to be an already given world of tools and concepts (Kisiel, 1985). Generational culture change provides another example of human creativity, as generations devise new meanings that are not transparent to earlier generations.

What does the metaverse make possible for individual and team creativity? The search for what’s familiar, we suggest, can trap teams and lead to the stunting of imagination. Creating the familiar can perpetuate real-world divisions and biases and can result in exclusion, marginalization, and segregated environments in which members work virtually (Richter and Richter, 2023). In the metaverse, users are able to adapt content, symbols, and various objects, such as their avatars. They can adapt the environment, their behavior in social relationships, and their knowledge. We propose further research into three areas of human experience potentially transformed by the metaverse: interaction, the self, and time. In the following section, we explore how these three aspects potentially affect team members in the metaverse.

We do not claim that the particular challenges and opportunities are *unique* to the metaverse but that they *cannot be ignored* in team coordination. We chose the three elements of interaction, the self, and time because they are important components of familiarity. Familiarity develops through time, through interactions, and in terms of identity or role. These aspects of fluid team functioning present creative opportunities and new challenges in the metaverse.

### 4.1 Interaction and fluid incorporation of “worlds”

The metaverse is a very social place (Hennig-Thurau et al., 2023) and requires interactional skills to establish a social presence. Such interactional skills can engender co-presence, focused interaction, and shared commitments (Schultze, 2014; Schultze and Brooks, 2019; Dincelli and Yayla, 2022). The skills entail not just language but also adaptation to a new embodiedness, with gestures and signals that transcend traditional physical and virtual distinctions.

The human body is an important repository of interactional knowledge, and this knowledge of the familiar is significant in devising ways of working in the new environments of the metaverse (Schultze, 2010, 2014). In unfamiliar settings, familiar ways to manage embodied and linguistic signals can be critical in achieving effective collaboration (Schultze, 2014) and effective TMS–even as they are adapted to the new setting. In globally dispersed interactions where informal interactions are reduced, engineers put reliance on formal asynchronous written means of recognizing, transmitting, and retrieving expertise (Jarvenpaa and Keating, 2011).

Enculturated behaviors play a role in managing shared expectations of others’ presence, allocating attention and being responsive, and converting intentions into actions with interlocking commitments. They influence the metaverse and shape how the body is transfigured or, according to Vidolov (2022), is “rematerialized” into a digital representation. The literature on virtual spaces often argues that virtual spaces entail a loss of the body (e.g., Vaast, 2007; Dincelli and Yayla, 2022; Nordbäck and Nurmi, 2023). In contrast, Vidolov (2022) points out that through tacit behaviors, the body in virtual environments actually is *extended* as the physical environment and technology merge to render interactions in virtual collaborations.

This extension process has its challenges. To illustrate, during COVID-19, workplace contexts for interaction and the experience of the body in interaction changed significantly as people had to assemble teams in largely unfamiliar, technology-mediated environments. Not only was the technology new, but people also had to be creative in adapting interaction. They found themselves in a suddenly unfamiliar work world; because of quarantines, this world had to exist both temporally and spatially in the very familiar environment of home, the children, pets, and delivery drivers’ knocking at the door. Members of work teams had to learn to behave in technology-mediated contexts and even to move rapidly between different contexts, familiar and unfamiliar, each with different participation frameworks and physical properties. To add to the challenge, people had to manage expectations about the fluidity between worlds–particularly when one world briefly broke unexpectedly into another.

During COVID-19, as in other unfamiliar contexts, people have developed new ways to use the body to signal co-presence in the online world and to manage co-presence. Follow-up research has shown that, even without a person’s image, tacit bodily work in a virtual environment can create three important aspects of successful interaction across teams--co-presence, readiness for focused interaction, and co-commitment (Vidolov, 2022).

First, to establish *co-presence*, people adapted their face-to-face habitual skills of perceiving whether a team member is available by checking onscreen icons, which became a form of gesture to signify whether someone was online and able to participate in work. Team members shifted the interpretation of whether “this person is available to talk with me” from the techniques they used in face-to-face interaction to new techniques that would work when they were geographically dispersed (Vidolov, 2022).

Second, to signal *focused interaction*, people adapted ways to convey attention, responsiveness, and their stance toward engagement, thus engendering a co-orientation signal beyond mere appearance. Here, a team member’s average response time and spontaneous engagements could be interpreted as bodily expressions and used to predict habituated and expected daily interactions.

Third, *co-investing* required a process of developing interlocking commitments between the self and the other; people managed by interpreting the timing and speed of others’ interactions (Vidolov, 2022). Yet the results were far from ideal, suggesting that less face-to-face interaction resulted in a reduced level of co-investment. In face-to-face interactions, people are used to noticing how they are being experienced by other people and can be seen as noticing this effect (Goffman, 1963), which creates an environment for co-investment.

As Merleau-Ponty (1968) observed, people experience themselves through interaction with others. Digital technologies challenge these processes because they “transform bodily senses in particular ways and rematerialize the body in a virtual context by transforming its capacity to express, sense, and be sensed” (Vidolov, 2022, p. 18). As one example, while at first people were frustrated by several people starting to talk at once in online team interactions, people devised a new turn-taking mechanism by using the mute/unmute icon on their own onscreen image to signal a desire to speak and interpreting that signal on others’ images similarly–much as an indrawn breath or eye gaze functions in face-to-face interaction. Adaptations take time, which is particularly challenging for fluid teams. Fluid team members lack the history of interactions from which they might draw new generalizations about the relationship between symbolic behavior and meaning–a relationship that, in turn, enables some predictability about a teammate’s actions.

Even Stephenson, the author of *Snow Crash*, acknowledged the challenges in managing human interaction in the metaverse. One of the story’s characters becomes wealthy by developing a system for instantly conveying embodied communication cues so the cues could be understood in a common way. However, Stephenson does not provide details about the system.

Research has shown that mutual attunement is fragile. For example, Vidolov (2022) studied a fluid team based in Ireland and described team members’ anxiety when a key member of the team, who was in India, was offline for long periods. In the second project the team undertook together, the collaboration broke down. “[I]gnoring even minor gestures can lead to disintegrational and relational breakdown,” notes Vidolov (2022: 19). Some challenges of the rematerialization process in the metaverse thus become clear.

How advanced virtual work spaces can accommodate the necessary communication for successful interaction may not be self-evident (Zhu et al., 2023). Language is used to establish particular relationships between things and people, including their hierarchy, interpersonal respect, and reciprocity. Yet, informal communication is needed to support such relationships. A study of collaboration in engineering design showed that shared visualizations were key to the support of joint acts to “discover,” “engage,” and “resolve” (Dossick et al., 2015). Settings characterized by high risk, stress, and time limitations (e.g., an operating room) also can prevent team members from achieving co-presence, focused interaction, and co-investing, particularly when some team members only passively watch, instead of interacting (Beane, 2019). And when a team includes artificial agents, additional challenges arise. Even when the goal of these agents is to reduce the human agents’ mental demand and effort of interacting in virtual environments (Xi et al., 2023), the high mental effort needed might limit human agents’ interactions with the artificial agents (Maedche et al., 2019; Seeber et al., 2020a; Zheng and Jarvenpaa, 2021).

Metaverse users face moral and professional questions about their identity extensions in interaction, such as their social standing and their control over their thoughts and judgments that might deviate from their expected occupational behavior. In collaborative work, a common experience involves finding one’s self to be “continually enlisted in others’ designs” (Holt, 2023, p. 8). In the metaverse, others’ designs are not only managers’ goal-directed projects, but also the environment itself embedded in complex machine-learning algorithms. Digital hate and other forms of harassment are already recognized as a major concern (Dwivedi et al., 2022; Rosenberg, 2022).

### 4.2 The extended self in the “worlds”

In considering the effects of the metaverse on fluid teams, the presentation of the self, or identity, is important. Individuals may adapt and extend their identities, including by accessing various possessions (e.g., digital possessions) and other agents (Belk, 2013). Sociologist Zygmunt Bauman proposed the term “liquid modernity” (Bauman, 2000) to describe the modern era, where identities are “irreparably fluid, ambivalent and otherwise unreliable” (Bauman, 1993, p. 234). The younger generation’s resistance to gender stereotyping, the global situation of increasing migration across cultures, and other factors have resulted in a heightened appreciation for what have been called fluid identity constructs (see, e.g., Boyd et al., 2020; Brown, 2022). The linkage of identity with place or space also has been undermined by or disconnected in social media spaces, where location is undetermined.

The metaverse makes new forms of self-possible. Avatars alter possibilities not only for the kinds of sensory experiences anchored in the body, but also for self-presentation (Schultze, 2011; Jin, 2012; Mirbabaie et al., 2021; Seymour et al., 2021). In a *BBC Three* report^5^ that looked at intimate relationships between avatars, a couple (Sam and Ryan) projected their identities as fictions in a virtual reality world, and within these fictions, they developed a relationship that led to marriage in the “real world,” although they had lived 5,000 miles apart. Ryan’s avatar identity consisted of a fox in human form, while Sam’s avatar had black hair that was punk styled. Ironically, through their fictional identity constructs, Sam reported feeling a more authentic sense of self and liked that she did not have to worry about how she looked, while Ryan also cited non-physical attractions. This comment suggests that the identities were not conceived of by the couple as separate but were fused or fluid. The type of social presence capability in the metaverse positively influenced these two interactants’ evaluations and emotions (see, e.g., Hennig-Thurau et al., 2023) and enhanced their feelings of positive self-identity.

Three challenges in relation to the self and the extended self-include the trustworthiness of avatars or representations of both self and others, maintaining control of personal goals and priorities, and navigating the increased learning potential as a self who is part-human and part-machine.

An avatar, as a self without all the sensory capabilities of the physical body, must find ways to establish trust–both in others and in the self. Avatar studies find that when the avatars resemble the user in physical appearance [e.g., in facial (eyes) and body (weight and height) characteristics], the user shows more engagement and more trust and loyalty (Suh et al., 2011). Familiarity in avatar faces and bodies also promotes social cohesion (see, e.g., Suh et al., 2011; Jin, 2012; Venkatesh and Windeler, 2012; Hooi and Cho, 2014; Seymour et al., 2018, 2021; Baek et al., 2022; Gräf et al., 2023; Kim et al., 2023; Litvinova et al., 2023). Familiarity increases the likelihood that behavior is similar in the virtual world and the real world (Dincelli and Yayla, 2022), and avatars that carry the social norms and expectations from the real world better regulate performance in the virtual world.

As people take on more complex and less familiar identities, they might feel out of control, lack confidence, or be confused about expected behaviors. Choosing a gender for their avatar that is different from the way they identify in the physical world may require learning an entirely new repertoire of behaviors. This learning impacts the development of TMS between the human and artificial agents. In an organizational setting, they might find few safe spaces for learning and experimenting with the self, unless such exploration is strongly endorsed by the leaders of the organization. As humans extend their bodies in the metaverse, they find that avatars can have sensory disadvantages that impact the sense of control. To draw attention to what’s lost in sensory cues, one character in *Snow Crash* asks another: “Can you make a little more noise when you walk?” (Stephenson, 1992, p. 126).

### 4.3 Time and work in the metaverse

Through centuries, new organizational forms have resulted from technology’s creating new possibilities for space–time configuration (Schultze and Boland, 2000). Fluid teams are simply a more recent example. The configurations of space and time determine available resources and how individuals adapt, and the metaverse is a new design space for such configurations (Seidel et al., 2022). The literature on fluid teams reports no consensus on how familiarity and time relate: Time as an antecedent to or influence on familiarity also is poorly understood in the team literature (Muskat et al., 2022).

The metaverse may bring about alterations not just in the nature of space–time but also to the organization of time. For example, an engineering team collaboratively building simulations in virtual space about future events in “real time” can reorganize time according to the requirements of the task or to correct mistakes made in the past (Dossick et al., 2015). For residents at the hospital tasked with using advanced robotics in operations–but with no opportunity to learn alongside senior physicians nor time in their regular physical cycles to practice–reorganizing time allowed them to undertake different occupational and role planning, to specialize much earlier, and to narrow the robotic skillsets they needed (Beane, 2019).

We consider three possibilities for time alterations: new conceptions of time (potentially leading to new experiences), new ways to map activities to time, and new relations between actors or team members and time (Ancona et al., 2001; O'Connor et al., 2023).

First, technologies have long been associated with *new conceptions of time*. In the 1950s, machine operators created a new social time, “banana time,” to increase playfulness in their work day and to reduce boredom in their repetitive work (Roy, 1959). Ivaturi and Chua (2023) coined the term “ubichronic time,” in which digital technology crams disparate activities into tiny units of time. Video recording technologies made it possible to view recorded time in faster or slower time rhythms, and organizational training videos often are viewed by advancing the speed. This ability can give the sense of moving “faster through time,” or it can lead to “found time” or even to “reclaimed time” that otherwise would be wasted (Ivaturi and Chua, 2023: 321).

In relation to the metaverse, Mogaji et al. (2023:1), discuss immersive time: “conscious, deliberate and dedicated time.” In the opening scene of *Snow Crash*, Hiro delivers a pizza on his smartwheels–a high-tech skateboard. The scene is a moment of intense focus and precision, as Hiro navigates the crowded streets with speed and agility. Time disappears in the flow. This immersive time can entail “escaping from,” and engaging in the metaverse can be seen “at least in part to escape the real world” (Mogaji et al., 2023, p. 1). To one writer, self-described as “a wheel-chair bound dwarf female” who is mobility restricted, her time in the metaverse offers “the sense of not quite being alone…. A lot of writers like to sit in cafes. I cannot do that, so I think it’s the next best thing. Also, the sounds are quite relaxing. And the simple mood-setting. And the routine of logging in and sitting in my nice, peaceful swamp. Getting in the right mood and frame of mind is terribly important, I think” (Davis and Chansiri, 2019, p. 500).

This type of immersive experience or “flow” can bring enjoyment: Immersive time can be seen as “escaping to” (Mogaji et al., 2023). People can be freed from their physical world barriers, such as physical imprisonment, marginalization, exclusion, or physical world barriers resulting from physical disabilities (Davis and Chansiri, 2019), as in the case of the wheelchair participant. However, immersive time also has a dark side (Mogaji et al., 2023). Too much immersive time as “escaping from” is associated with metaverse addiction, where people become isolated from their social relationships and may become vulnerable to “digital slavery” (Mogaji et al., 2023, p. 3).

Whether escaping from or escaping to, the metaverse can create new views and conceptions of time for members and teams. New views create new possibilities. Although these new conceptions, experiences, and senses of time are initially unfamiliar, they become familiar through repetition and routines.

A second aspect of time alteration emerges from *new ways of mapping activities to time*. Participating with others in video games involves a rather deliberate “time design”; game designers exploit the game features, norms, routines, and expectations to curate different temporalities at individual and collective levels to regulate player engagement (Rapp, 2022). Fluid teams that are global require team members to synchronize their activities across time zones to a shared time vision (Saunders et al., 2004). However, synchronization can create asynchronies in relation to local routines (e.g., holidays; Jarvenpaa and Keating, 2011), as well as extremely long work hours (Blagoev and Schreyögg, 2019).

Mapping activities to time may entail variations in how activities are perceived as dispersed through time, as well as how their duration or continuity through time is marked. For activities to continue in virtual environments, the environment needs constant attention and care. In the physical world, objects and people visibly age and wear over time, and natural processes, such as erosion and weathering, can alter their appearance and state. Beginnings and endings can be predicted or estimated based on the current state. However, in virtual environments, time is fluid and less visibly passing, so that the temporal patterns may not be predictable. Events that would take hours or days to occur in the physical world can happen almost instantly in the metaverse. For an activity to continue and then to be closed through linguistic gestures (e.g., “thanks for sending this so quickly”) constant care and attention is needed (Vidolov, 2022).

Agents in the metaverse can map time and activities differently by slowing time down or speeding it up. Virtual manipulation of time and space is possible through software coding, creating new possibilities for actors to experience time. For training purposes, real-time simulations might aim to replicate real-world time, and virtual objects might be programmed to age and change over time, just as they would in the real world.

Time is likely to be an important metaverse design feature that allows users to manipulate time by setting their own preferred times. Fluidity of time in virtual environments allows fluid team members to manipulate time in unconventional ways. Some manipulation is evident in the ways that individuals purposively split a single block of time into multiple discontinuous time series or change schedules on the fly (Ivaturi and Chua, 2023). Yet, these individual time manipulations can create difficulties for team synchronization or a shared time vision. Constant changes in rhythms, frequencies, or intervals of activity (e.g., interaction or task completion) can lead to disintegration of communication unless external leaders undertake interventions to explain them (Vidolov, 2022).

Manipulating time is a form of power in *Snow Crash*. When Hiro is being chased by Raven, he uses his knowledge of the metaverse to create a custom time zone that allows him to move more quickly and evade his pursuer. When Hiro encounters the Librarian, he experiences a moment of profound insight and understanding of the underlying patterns and structures that govern the universe. The concept of “mu,” referred to as emptiness or nothingness, helps Hiro to clear his mind and perceive reality in a new and profound way; thus, he overcomes various illusions of the environment (guards and security measures) and infiltrates the virtual headquarters of the antagonist.

A third aspect of time to consider is *agents’ relations to time*. The metaverse is likely to alter actor–time relations. Yet, the fictional work of *Snow Crash* exhibits rather conventional distinctions in agents’ temporal focus (short term versus long term). Juanita, one of Hiro’s allies, is a highly educated and successful programmer, and her success reflects her ability to think about time in a longer term way and to make decisions that will benefit her and the society in the future. Her actions exhibit ways in which manipulating perceptions of and relations to time could shape experiences of time to create better futures for individuals and societies.

*Snow Crash* assumes the linear and continuous relationship of past, present, and future as in a traditional narrative, and knowledge is maintained by telling stories that use this sequential pattern in both physical and virtual worlds. Stories allow agents to be reflexive, and protagonist Hiro references the poet, Muriel Rukeyser, in observing that the perception of reality is shaped by the stories we tell ourselves and each other, rather than by any facts.

What implications does the ability to alter time in these three ways–in its conceptualization, its mapping to activities, and the human/time relationship–have for one of the crucial mechanisms used to coordinate collaborative work? That is, what do they mean for the effectiveness and speed of developing and updating TMSs– of identifying who knows what? In the physical world, TMS development depends on communication and coordination of distributed expertise and validation of expertise (Brandon and Hollingshead, 2004). TMSs assume a cyclical but linear process – knowledge cannot be validated before it is communicated; this process reflects the interdependencies (both sequential and reciprocal relationships) in task completion and in the roles of a fluid team.

In contrast, in the metaverse, transport across vast spaces or processing of huge amounts of information can be achieved instantly. Hence, team members may face failures as they try to adapt in the metaverse to a conventional rhythm or cycle of time. The notion of “updating” in the metaverse may be meaningless if present interactions are automatically recorded and immediately become part of a permanent history. In the metaverse, conventional linearity and ordering may no longer prevail as a shared principle. Sequences may not be preprogrammed, and members may have full autonomy for action in complex and fast-paced tasks. There may not be any external leaders to determine or regulate order through schemas or scripts.

The fleeting presence of members in fluid teams may mean that their participation and contributions largely go unrecognized by other members and by later iterations of the team unless dedicated attention is devoted to making time concrete and specific. Team membership assumes that at some marked time, members join, fulfill their tasks, and leave. Creating shared agreements of time, and hence membership, may not be possible.

## 5 Research directions and practical implications

**Figure 1:**
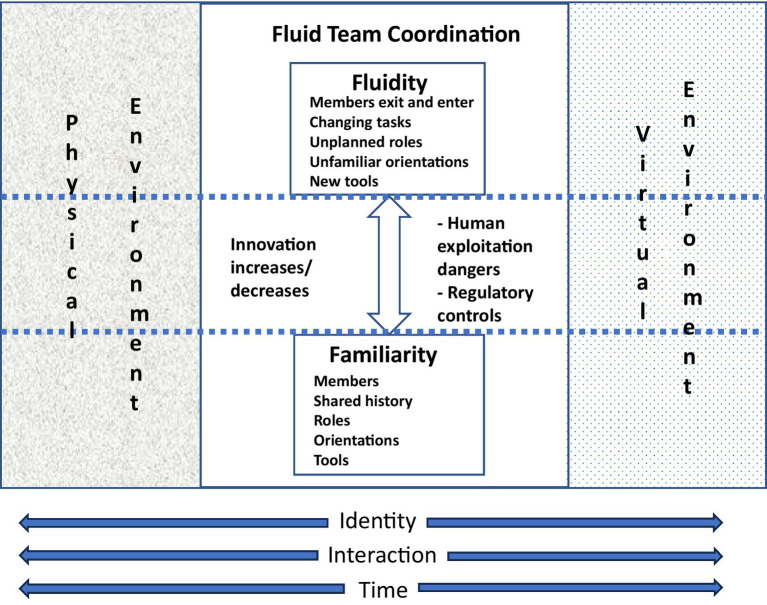
Fluid organizational teams and familiarity in the metaverse.

In this work, we considered the importance of familiarity in fluid teams and in the context of the metaverse, and we explored interaction, “the self and relationships (identity), and time in these contexts” (see Figure 1). Interaction is positioned between identity (the self and relationships) and time in Figure 1 because the latter two are influenced by interaction.

Next, we describe five potential research paths to advance understanding of familiarity as it relates to fluid teams and the metaverse, along with possible practical implications (see Table 1).

**Table 1:** Research paths and practice implications for “hype, hope, or hell”.

Research path	Potential research questions	Practice implications
Relationship of fluidity and familiarity in organizational teams in the metaverse	How do fluidity and familiarity mutually influence each other for both human and non-human agents in team coordination? How does their influence vary at different levels of fluidity and familiarity in members and the environment? How does their influence affect coordination for innovation? How does the relationship between fluidity and familiarity affect performance, in cases of both incremental and radical changes in the levels of fluidity and familiarity? (How are members’ contributions recognized over time? How can communication be enhanced?) In what ways are the costs of familiarity increased when fluidity changes rapidly?	Engage in gradual increases in fluidity to diminish the high costs of increasing familiarity. Identify how new members should be trained (e.g., mentorship with humans or AI-driven avatars). Determine ways to harness creativity that emerges from unfamiliarity in the metaverse.
Relationship between virtual and physical environments in the metaverse	How do the decisions and actions of fluid teams affect their integration, parallel management, and/or fusing of the physical and virtual environments? How do fluid team members in the metaverse experience virtual and physical worlds, both individually and in relation to each other? (For example, does work time expand, are interactions satisfying, how are identities managed?)	Leverage virtual environments for the virtual potential and physical environments for the physical potential, rather than importing familiar members, tasks, roles, and tools from physical to virtual environments, or vice versa, just because they are more familiar.
(Un)familiar interacting in fluid teams in the metaverse	How and how rapidly does interaction (e.g., language, gestures, and signals) evolve for fluid traversal between physical and virtual environments in the metaverse? How will the human body’s socialization to interactional routines adapt to fluid worlds and how might it juxtapose for symbolic or communicative purposes the (stark) differences that remain?	For the near future, translate and skillfully use scripted and habituated language, gestures, and signs, drawn from physical and virtual environments, in the metaverse for co-presence, co-focusing, and co-investing. Watch for technological breakthroughs (e.g., AI tools) and environ-mental jolts (COVID-19) that can potentially transform, not just translate.
(Un)familiar identity in fluid teams in the metaverse	How does (un)familiarity among members in the physical environment affect (un)familiarity in members’ extended identity representation in the virtual environment? How do AI avatars change the fluidity of member identity representation in physical and virtual environments? What are the implications for team-level behaviors (e.g., stereotyping)? What protective mechanisms are needed for extended and fluid identity representation to avoid social injustices (e.g., marginalization or hate speech)?	Experiment with protective mechanisms for fluid identities to avoid injustices and address moral dilemmas. Identify ways to manage the human propensity to anthropomorphize other forms of life and objects.
(Un)familiar time in fluid teams in the metaverse	How can time be altered in the metaverse to increase, not diminish, the power of members? What are the implications for teams? When time is manipulated in the metaverse, how can teams coordinate cultural conventions (e.g., time at work vs. time with family) across fluid boundaries? How can the relationship between time spent and familiarity achieved (e.g., with roles or tools) be measured? How can continuity through time be achieved?	Use metaverse to experiment with time and to identify new ways of interacting. (Team coordination may deteriorate.) Consider new ways to account for work time to prevent burnout.

The first research path explores the relationship of fluidity and familiarity in fluid teams in the metaverse. Here, we consider how fluidity and familiarity are experienced by organizational teams in the metaverse. In physical environments, we know that coordination between team members who are (or feel they are) strangers to each other is facilitated by the meso level structure of roles that have clear boundaries and accountabilities. We also know that familiarity in peer relationships (e.g., peer physicians) affects people’s willingness to multitask and redistribute tasks: “I’ll take this patient if you take the next” (Niewoehner et al., 2023, p. 958). Repeated interaction between peers increases trust and cohesion, and leads to a higher work effort by individuals. Higher trust and cohesion can absorb uncertainty, and members may be more willing to accommodate instability. As a result, familiarity itself can fuel fluidity, and vice versa. This type of mutual influence on relationships is likely to exist not just in physical environments but also virtual environments.

Yet, in terms of future research, much still needs to be learned about how fluidity and familiarity mutually affect each other at a particular level, such as a member or team, and also at a cross-level in the metaverse. In the metaverse, both familiarity and fluidity are likely to be highly dynamic and to shape and be shaped by ever-changing permutations of the contributors to familiarity and fluidity (e.g., members, tasks, roles, orientations, tools). How changes in familiarity and fluidity unfold likely matters as well. How might incremental versus radical changes in familiarity and fluidity affect their relationship in terms of team coordination? Familiarity might come with substantive costs with high levels of fluidity.

A second research path examines the relationship of virtual and physical environments in the metaverse. Different writings on the metaverse depict different relationships between virtual and physical environments. Some studies emphasize the two as interconnected experiences in which virtual environments dominate these experiences (Seidel et al., 2022). Some see physical and virtual environments as parallel worlds (Marabelli and Newell, 2022), and others construct them as indistinguishable (Whang and Miranda, 2023).

We suggest that future research must delve into the mutuality of the relationships between physical and virtual environments in the metaverse. How a particular organizational team experiences the virtual and physical worlds in relation to each other is likely to lead to different team goals and to differences in the resources needed in the environments. For example, the two environments may vary greatly in terms of what tools are available. In terms of practice, that virtual environments are leveraged for the virtual potential and that physical environments are leveraged for the physical potential is important. In developing practices, we suggest the need to avoid imposing scripts, tools, and times from physical to virtual environments, and vice versa.

The third research path encourages research on language, gesture, and signals for fluid traversal between the physical and the virtual environments of the metaverse. For fluid team members in organizations, watch for a strong preference to establish that one’s virtual representation (e.g., avatar) is indeed oneself–that is, “…a user’s sense that her avatar’s appearance and (scripted) gestures, as well as the attitude and impression they give off, are her own” (Schultze, 2010, p. 438).

Future research also can help to improve our understanding of how artificial agents may change the dynamics of interaction and the role of body. Such studies need to go beyond dyadic human–AI interaction to consider team-level behaviors. Moreover, little may change in interaction if artificial agents are merely seen as extensions of a self (Mirbabaie et al., 2021) and not as having a presence of their own. Even as organizations seek more flexible virtual forms of organizing through new space–time configurations, the body still matters in the workplace of the future (Nordbäck and Nurmi, 2023). Understanding the body in interaction is important for team coordination and for managing risks for individuals and organizations, including risks to security (e.g., technological identity threat) and privacy (Falchuk et al., 2018; Mirbabaie et al., 2021; Gräf et al., 2023; Kumar et al., 2023).

The fourth research path addresses the constructions of the self and relationships that rely on the familiar to instill trust and control and to enable learning. The ways in which people continue to mindfully replicate their own characteristics from the physical world (e.g., their shape and gender) in the virtual world is surprising. Even changes in clothing appear to mirror patterns from the physical world. Morality and rationality are transported from the physical world because users see their virtual presentations as having repercussions in the physical world. As already noted, a “wheel-chair bound dwarf female” gave up on an avatar depicting a tall and thin western idealized young woman: It simply “did not feel right” (Davis and Chansiri, 2019, p. 499). She changed the avatar to a whimsical reptilian animal form.

Future research needs to examine how avatar representations change when avatars are technologically controlled rather than human controlled. Moreover, how is the locus of control represented, and how do these representations influence team members or team behaviors? Agency and morality concerns also require research to ensure protections and safeguards that avoid social injustices.

The fifth research path concerns time. Time might be the factor by which fluid teams have the best opportunity to depend less on the familiar and on physical and virtual congruity. Although we have suggested a few opportunities, behavioral fidelity again may hamper both innovation and a realization of the potential of virtual worlds to free humans from ordinary constraints. Perhaps not surprisingly, postings on various forums suggest feelings of subordination by technology: “I spent my entire life gaming and I feel useless”.^6^

One conception that fascinates and deserves future research is the time-travel feature of the metaverse. Such a feature would allow traveling to the past and to the future. Being able to experience the economic, social, and moral implications of decisions in a distant future can render more meaningful discussion of decisions being made in the present. Can the metaverse improve responsible engagement and accountability in the climate crisis? Will the metaverse obscure and obliterate time and make a persistent commitment even harder to get in the long term? In terms of practice, time might be the new wild ride in the metaverse, with consequences – particularly in terms of team coordination–difficult to predict.

In terms of time, interaction, and identity, we call for research that goes beyond the “intuitive” designs that seek to recreate the familiar in terms of user experience (e.g., providing a doorway to nudge a user toward a particular next action). Critiques of virtual reality commonly focus on whether the virtual world is realistic compared to “real life.” Research studies are set up to produce agents that are reliably similar to how humans behave; they impose temporalities that conform to existing power structures. Overall, we call for research that leads to understanding of the fluidity in participation between the metaverse and other “worlds,” even as we recognize that metaverse experiences are not forecasted to completely replace already familiar interactions. Our point is that if the “new, new thing” is created in mimicry of the familiar, or if it is evaluated only in relationship to the image of the old, this perspective effectively limits the scope of change and possibility.^7^ To combat such limitations, some observers already call for an emphasis on the unfamiliar rather than the familiar: “Organizations should start now to incorporate dynamic AI agents that can begin their entry into the next phase of connectivity *to reshape the way we interact*” (see footnote 7, respectively).

## Author contributions

SJ: Writing – original draft, Writing – review & editing. EK: Writing – original draft.

## Footnotes

^1^Note that in this paper we use “worlds” and “environments” interchangeably.

^2^https://youtu.be/gElfIo6uw4g

^3^Producing artificial agents that are reliably similar to how humans behave ignores the phenomenon of constantly changing and emergent forms of human interaction and perhaps only ensures that the goal of creating interactants-like-humans can never quite be met (Holt, 2023).

^4^https://www.forbes.com/sites/forbestechcouncil/2022/07/11/into-the-metaverse-the-future-of-virtual-interactions/

^5^https://www.bbc.co.uk/bbcthree/article/d41b2883-a690-443d-b663-2bd5bf007ccb?xtor=ES-208-%5B61744_NEWS_NLB_GHI_Wk23_Fri_9_Jun%5D-20230609-%5Bbbcthree_popmusiclizzolifestory_bbcthree%5D

^6^Redditr/StopGaming, 2018.

^7^https://www.forbes.com/sites/forbestechcouncil/2022/07/11/into-the-metaverse-the-future-of-virtual-interactions/?sh=5e14b6ab4cb5
